# Mating status correlates with dorsal brightness in some but not all poison frog populations

**DOI:** 10.1002/ece3.3531

**Published:** 2017-11-01

**Authors:** Corinna E. Dreher, Ariel Rodríguez, Molly E. Cummings, Heike Pröhl

**Affiliations:** ^1^ Institute of Zoology University of Veterinary Medicine Hannover, Foundation Hannover Germany; ^2^ Section of Integrative Biology University of Texas Austin TX USA

**Keywords:** brightness contrast, color divergence, dendrobatid frogs, mate choice, sexual selection, visual modeling

## Abstract

Sexual signals are important for intraspecific communication and mate selection, but their evolution may be driven by both natural and sexual selection, and stochastic processes. Strawberry poison frogs (*Oophaga pumilio*) show strong color divergence among populations, but coloration also varies among individuals of the same population. The importance of coloration for female mate choice has been studied intensely, and sexual selection seems to affect color divergence in strawberry poison frogs. However, the effect of coloration on mating success under field conditions has received very little attention. Furthermore, few studies examined how phenotypic variation among individuals of the same color morph affects mate selection under natural conditions. We measured the spectral reflectance of courting and noncourting individuals and their background substrates in three geographically separated populations. In one population (Sarapiquí, Costa Rica), we found that naturally occurring courting pairs of males and females had significantly brighter dorsal coloration than individual males and females not engaged in courtship interactions. Our field observations suggest that, in the wild, females prefer brighter males while the reason for the higher courtship activity of brighter females remains unclear. Overall our results imply that brightness differences among individuals of the same color morph may actually affect reproductive success in some populations of strawberry poison frogs.

## 1 INTRODUCTION

Sexual signals such as mating calls, color patterns, or behavioral displays are important for intrasexual communication and mate selection (Gerhardt & Huber, 2002; Setchell, Charpentier, Abbott, Wickings, & Knapp, 2009). The evolution of these traits may be driven by sexual selection, but may additionally be shaped by natural selection and stochastic processes, for example, genetic drift (Coyne & Orr, 2004; Richards‐Zawacki & Cummings, 2011). The strength of these forces depends on local conditions, hence geographically isolated populations are likely to differ in their sexual signal phenotypes (Gerhardt & Huber, 2002; Pröhl, Willink, & Hauswaldt, 2013; Rudh, Rogell, & Hoglund, 2007; Stuart‐Fox & Ord, 2004; Uy, Moyle, & Filardi, 2009). Variation in sexual signals combined with preference functions for these signals can lead to a Fisherian runaway process, causing rapidly diverging traits and accelerated speciation (Fisher, 1930; Iwasa & Pomiankowski, 1995). However, intraspecific variation in sexually selected traits is not only expected among populations, but also within populations. Variation in sexual signals can correlate with mate condition, mate quality, and/or serve as a means to exploit perceptual biases of the opposite sex (Ryan & Cummings, 2013; Ryan & Keddyhector, 1992; Zahavi, 1975).

Coloration and brightness of color patterns are used in intraspecific communication, and are known to influence mate choice in a variety of taxa (frogs: Maan & Cummings, 2009; fishes: Rowland, Baube, & Horan, 1991; mammals: Setchell et al., 2009). In amphibians, variation or changes in coloration have been mostly explored in the context of predator avoidance, that is, aposematism or camouflage as opposing ecological strategies, and thermoregulation (Maan & Cummings, 2012; Rudh, Breed, & Qvarnstrom, 2013). Recently, more studies have been added to assess the importance of visual, often colorful signals for conspecific communication during reproduction (Gomez et al., 2009). Neotropical poison frogs of the family Dendrobatidae are highly variable in coloration among species and populations (Lötters, Jungfer, Henkel, & Schmidt, 2007; Pröhl et al., 2013; Willink, Brenes‐Mora, Bolanos, & Pröhl, 2013), and to a lesser degree among individuals within a single population (Brusa, Bellati, Meuche, Mundy, & Pröhl, 2013; Richards‐Zawacki, Yeager, & Bart, 2013). A large body of knowledge has accumulated on visual signaling and other ethological aspects of these frogs currently used as a model system to study the evolution of phenotypic diversity in sexual signals (Cummings & Crothers, 2013; Rojas & Endler, 2013).

The strawberry poison frog (*Oophaga pumilio*; Schmidt, 1857) is highly diverse in coloration patterns among populations, with more than 15 different color morphs, mostly located in the Bocas del Toro Province of Panama (Daly & Myers, 1967). Natural selection may have promoted the evolution of color polytypism in this species (Maan & Cummings, 2012; Pröhl & Ostrowski, 2011), but sexual selection, geographic isolation, and genetic drift are also expected to have played a role (Cummings & Crothers, 2013; Gehara, Summers, & Brown, 2013). Strawberry poison frogs have color vision (Siddiqi, Cronin, Loew, Vorobyev, & Summers, 2004) and the females of several populations show color‐assortative mate preferences (Maan & Cummings, 2008; Reynolds & Fitzpatrick, 2007; Richards‐Zawacki, Wang, & Summers, 2012; Summers, Symula, Clough, & Cronin, 1999) and in some populations frogs show directional inter‐ and intrasexual selection for bright coloration in males (Crothers & Cummings, 2013; Crothers, Gering, & Cummings, 2011; Maan & Cummings, 2009). On the Panamanian Island Solarte, strawberry poison frogs display sexual dimorphism in coloration, presumably facilitated through female preference for brighter males (Maan & Cummings, 2009). The above‐mentioned studies investigated female choice in *O. pumilio* under laboratory conditions, and discovered general preference functions, which might underlie mate choice decisions. However, preferences found under laboratory conditions may not completely reflect natural choice situations. It is largely unknown whether (1) observed preferences translate in actual choice during courtship and (2) whether reproductive behavior observed under artificial laboratory conditions equals the behavior in the wild where it should be modulated by additional factors and costs related to mate searching (Meuche, Brusa, Linsenmair, Keller, & Pröhl, 2013). For example, in a captive breeding experiment, males and females from different populations and color morphs reproduced as successfully as within‐population pairs (Dugas & Richards‐Zawacki, 2015). Even though in this study, the frogs were situated in a no‐choice situation, it demonstrates that females accept nonassortative partners when no color‐assortative partners are present. Up to now, only one study conducted behavioral observations of natural, not manipulated mating behavior in the field and confirmed dorsal color‐assortative mating in a mainland population in Costa Rica (Gade, Hill, & Saporito, 2016). Thus, research investigating natural mate choice under field conditions is still scarce (Dreher & Pröhl, 2014; Gade et al., 2016; Richards‐Zawacki et al., 2012), and is explicitly addressed here.

Populations of the strawberry poison frog form two genetic groups, a northern group (Nicaragua and North Costa Rica) and a southern group (South Costa Rica and Panama; Hagemann & Pröhl, 2007; Wang & Shaffer, 2008). Despite significant population structure, both groups are connected by high levels of gene flow (Hauswaldt, Ludewig, Vences, & Pröhl, 2011; Rudh et al., 2007). In the northern group, frogs are predominantly red, while color polytypic populations are mainly found in Panama. Differences in coloration are generally found among different localities while the sexes do not or only slightly differ in coloration within local populations (Maan & Cummings, 2009; Rudh et al., 2007).

For this study, we selected three populations of strawberry poison frogs which cover the different genetic groups, and geographically span most of the species range (North Costa Rica, South Costa Rica and Panama). Hence, the frogs at our study sites differed in genetic affiliation, but also in color morph, evolutionary history, and probably toxicity (Saporito et al., 2007). The population at Sarapiquí (North Costa Rica) belongs to the northern genetic group and the frogs are red with blue legs (Hagemann & Pröhl, 2007; Wang & Shaffer, 2008; : Sarapiquí is here called La Selva). The population in Hitoy Cerere (South Costa Rica) belongs to the southern genetic group and the frogs are red with small dark spots; the population on Isla Colón in Bocas del Toro (Panama) also belongs to the southern lineage and is green with large dark spots. Phylogenetic analyses suggest that the Bocas del Toro clade resulted from recent and rapid population expansion of a lineage from south‐eastern Costa Rica (Gehara et al., 2013; Wang & Shaffer, 2008). This area was also identified as the place of origin of the species (Galindo‐Uribe et al., 2014).

Strawberry poison frog populations differ largely in the composition of their toxic alkaloids (Saporito et al., 2007). Combining coloration measurements, visual modeling and mouse‐based toxicity assays, Maan and Cummings (Maan & Cummings, 2012) detected a positive correlation between dorsal (but not ventral) color conspicuousness or brightness with toxicity across eleven frog populations in the Bocas del Toro area especially for bird predators. The conspicuousness or brightness of the frogs can therefore be considered as honest signal reflecting the ability to deter avian predators. Even though the toxicity of the Costa Rican populations is unstudied, the study suggests the green and more cryptic frogs are less toxic than frogs from the more conspicuous red populations (Maan & Cummings, 2012). The divergence in coloration among population has a genetic basis (Summers, Cronin, & Kennedy, 2004). However, it remains unclear whether variation among individuals of the same color morph in one population is genetically or environmental influenced, including potential plasticity in coloration depending on changing ecological or seasonal conditions.

Our investigation aims to explore whether evidence for sexual selection exists in three different natural populations of strawberry poison frogs by examining the color phenotype of reproductively engaged individuals (a courtship pair) relative to individuals not participating in reproductive interactions under natural field conditions. Due to the accumulated knowledge on the reproductive ecology of these frogs mentioned above, we concentrate on differences between populations, the sexes, and dorsal and ventral coloration. Specifically, we explore if males and females of *O. pumilio* observed in pairs and engaged in reproductive activities (i.e., courtship but not amplexus) differ in brightness and coloration from single individuals not engaged in courtship activities. In strawberry poison frogs, reproductive success of males seems to be directly correlated with the number of successful matings (i.e., laid clutches), unless clutch loss is extraordinarily high (Pröhl, 2005). Therefore, differences in the color phenotype of courting and single individuals in a sampled population could be indicative of differences in the mating success of these phenotypes.

## 2 MATERIALS AND METHODS

### 2.1 Field work

Spectral reflectance of the skin of male and female strawberry poison frogs was measured under controlled conditions in each study population. Individuals were classified as courted (=“courtship”) or isolated (=“single”), based on contextual observations. Frogs classified as “single” were haphazardly selected and observed for a couple of minutes to assure they were not courting at this particular moment. Obviously, these frogs might have been involved in courtship activities before and after; hence “single” might not represent a general mating status. With this method, we might risk masking existing differences between reproductively active and nonactive animals. However, it is a conservative approach because any significant results will provide evidence of a true difference in coloration between “courted” and “single” animals. Pairs of frogs found in the field were observed until courting behavior was undoubtedly identified. In an early stage of the prolonged courtship of strawberry poison frogs (C. E. Dreher, personal observation), males are quite mobile and call to females in their proximity, while the females usually do not show any visible response to male courting behavior. This early stage of courtship can take up to 1 hr or more (C. E. Dreher, personal observation). Later during courtship, the female starts to react to and approach the calling male. Alternately emitting advertisement calls, hopping and walking, the male will lead the female toward the oviposition site. We considered a female to have made a decision to mate with the calling male once she was observed to follow him. Courtship in this ultimate stage is rarely interrupted and normally ends in clutch deposition below leaf‐litter (Meuche et al., 2013; Pröhl & Hödl, 1999). Prior to the pair entering leaf litter, we captured the couple in order to avoid losing the frogs. Captured courted and single frogs were stored individually in perforated plastic containers with a moist tissue and several leaves. Reflectance measurements were taken in the afternoon, usually within eight hours after capture, under standardized handling procedures and light conditions. In each study population, between 39 and 52 frogs were measured (Table 1).

**Table 1:** Study sites and number of frogs (*N*) for behavioral observations and spectral measurements

Study site	Geographical coordinates	Habitat	Courted/single males (*N*)	Courted/single females (*N*)
Sarapiquí	10°28.227′N; 84°0.553′W; 44 m.a.s.l.	Mix of young secondary forest with old cacao plantation	9/10	10/10
Hitoy Cerere	9°37.819′N; 83°0.879′W; 270 m.a.s.l.	Mix of young secondary forest with old banana plantation, many palms	10/10	10/10
Isla Colón	9°23.170′N; 82°15.941′W; 35 m.a.s.l.	Old secondary forest, closed canopy, many *Heliconia* & *Dieffenbachia*	13/13	13/13

Additionally, we measured the reflectance of the substrate (e.g., leaves, trunks) on which each frog was found. Reflectance spectra of the skin or substrate were taken at a distance of 2 mm using an Ocean Optics bifocal optic fiber (R‐200‐7‐UV/VIS) with a fixed outer sleeve to control the 2 mm distance, an Optics HR2000+ Spectrometer, and a deuterium–tungsten lamp (DT‐Mini‐2‐GS). To account for lamp drift, we calibrated the measurements with a white standard (WS‐1‐SS) every other frog. Illumination of the habitat (Irradiance) was measured using an optic fiber (QP400‐2‐UV‐BX) with an Ocean Optics cosine adaptor‐head (CC‐3UV) attached. Irradiance spectra were taken at the places where we found the frogs and at times when the frogs are reproductively active (between 7 a.m. and 12 p.m. in Costa Rica; between 8 a.m. and 1 p.m. in Panama) on two to three different days. The population‐specific average irradiance was calculated for each population, using between 240 and 396 irradiance spectra per population.

Dorsal reflectance spectra were calculated averaging four reflectance measurements (two of which were taken on the head between the eyes, and two on the middle of the dorsum). Calculations of dorsal average spectra for frogs from the population on Isla Colón, where frogs possess a dark spotting pattern on a green background color on their dorsum, included two measurements of the green background color and the two head measurements. Ventral average spectra in all populations were calculated averaging two reflectance curves taken on the belly. We did not include measurements taken from the throat region in order to avoid the darker coloration of the throat of males to impact the results.

Visual models were calculated according to Maan and Cummings (2012) and Crothers and Cummings (2013) using average dorsal and ventral reflectance spectra from each frog, the population‐specific average irradiance and microspectrophotometric data on the visual sensitivity of cones of *O. pumilio* (Siddiqi et al., 2004). For the calculation of brightness contrast (ΔL) and color contrast (ΔS), we additionally included reflectance spectra of the individual‐specific substrate for each frog. In addition, to account for variation in conspicuousness driven by an animal's specific background rather than the inherent reflectance properties of its body, we calculated a color (S) and brightness (L) value for each frog independent of background that included only frog reflectance spectra, the population‐specific average irradiance, and data on visual sensitivity of *O. pumilio*. The (S) and (L) values indicate the intrinsic conspicuousness, that is, a value that indicates how well the frogs` coloration can be detected by the visual system of the respective observer. For comparison of the overall brightness of individuals, we calculated total reflectance flux, summarizing the recorded reflectance for each nm (ΣR_(λ)_ for λ = 300–700; Crothers & Cummings, 2013; Maan & Cummings, 2012).

The study was conducted in accordance with German, Costa Rican, and Panamanian laws and followed the “Guidelines for the treatment of animals in behavioural research and teaching” (2001) and the “Guidelines for use of live amphibians and reptiles in field research” (1997).

### 2.2 Data analysis

Measurements of the dorsal and ventral spectral reflectance variables (ΔL, ΔS, L, S, ΣR_(λ)_) were subjected to separate principal components analysis (PCA) and the individual scores of the components with eigenvalues <1 were retained for subsequent analyses. This strategy reduces the dimensionality and collinearity in the original variables while maintaining separate dorsal and ventral PC‐derived spectral variables that facilitate biological interpretation of results. Homogeneity of variance of PC scores across levels of the predictors was confirmed with Levene tests. We tested the effects of three categorical predictors: locality (Sarapiquí, Hitoy, and Isla Colón), mating condition (courted/single), and sex (male/female) on the spectral reflectance values with a multivariate analysis of variance (MANOVA) using the principal components as dependent variables and allowing for interactions among predictors. Statistically significant multivariate effects (α* *= 0.05) were also inspected with univariate analyses of variance, and significant effects were explored further by plotting the 95% confidence intervals of the means of the PC‐derived spectral variables, after grouping the data into predictor categories, and with post hoc pairwise comparisons using the Tukey test. All statistical analyses were conducted with R (R Core Team, 2016).

## 3 RESULTS

**Figure 1:**
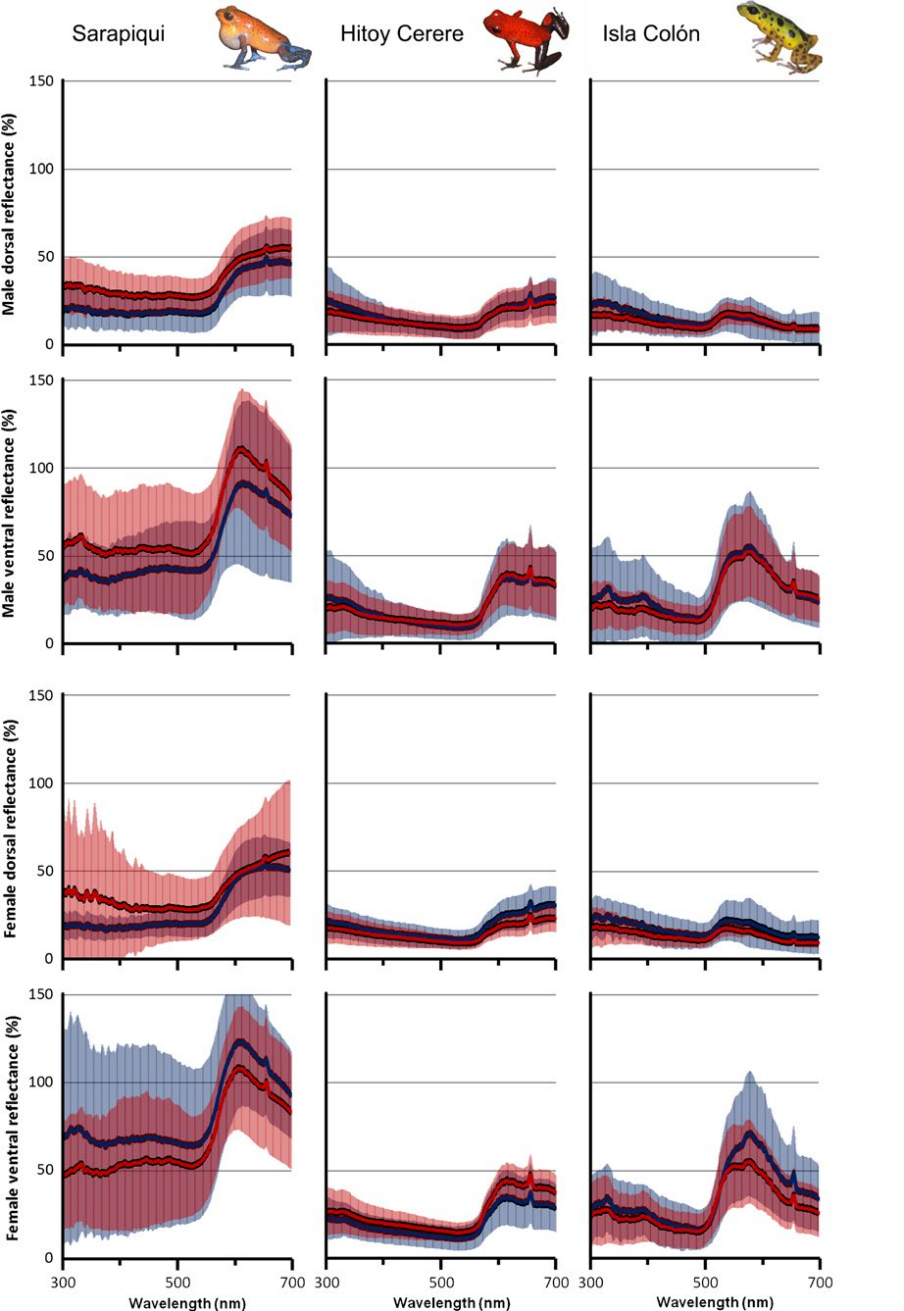
Mean reflectance spectra (in % reflection relative to white standard) including standard deviation of mated (red line) and unmated (blue line) individuals of *Oophaga pumilio* from three study populations. Male and female, as well as ventral and dorsal reflectances are shown in separate graphs. Mean reflectance curves average spectra of nine to 13 individuals (Sarapiquí males mated (dorsal and ventral): *n* = 9; for all Isla Colón spectra: *n* = 13, for all other spectra: *n* = 10). Reflectances over 100% emerge due to the brilliant surface of the frogs’ skin, while the used white standard has a dull surface. In order to verify accuracy of reflectance curves, all spectra were visually controlled for oversaturation

Mean spectra of females and males from all three populations are presented in Figure 1. Generally, the dorsal and ventral spectral reflectance was highest for frogs from Sarapiquí; and in this population, differences between “courted” and “single” frogs are more pronounced than in the other two populations (see below).

**Figure 2:**
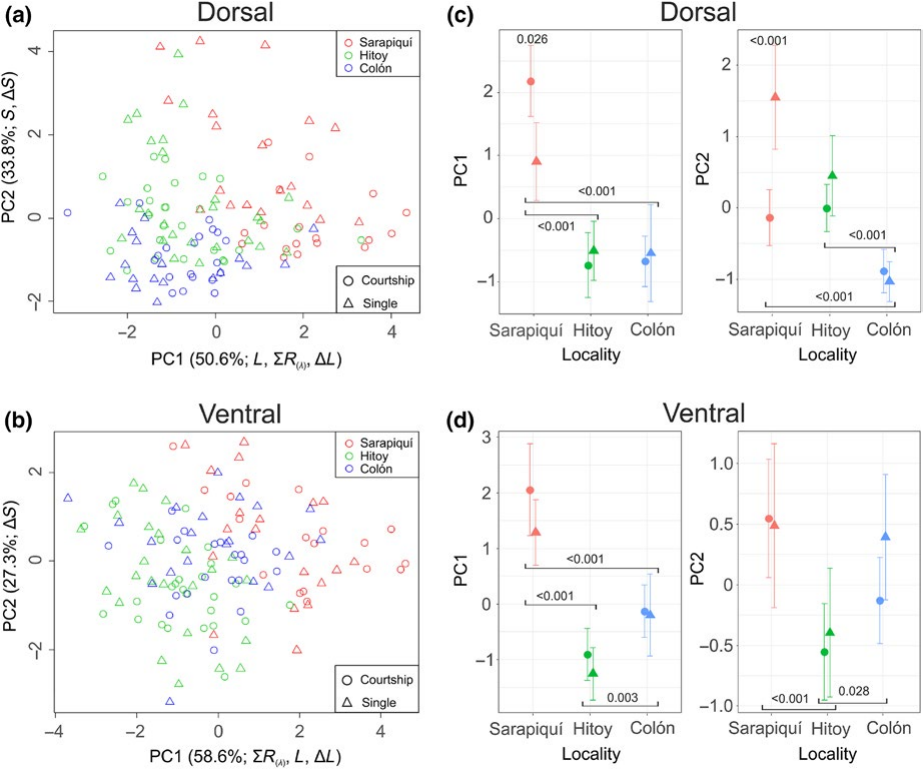
Variation in spectral variables of 128 individuals of *Oophaga pumilio* derived from PCA of dorsal and ventral spectral measurements. Plots on the left show the individual scores of the first two components extracted from five dorsal (a) and five ventral (b) spectral variables with symbols and colors identifying the corresponding locality and observed mating condition; the percent of explained variance and most contributing variables (Pearson's *r *>* *.7) are indicated on the axes. Panels c and d illustrate the means (symbols) and 95% confidence intervals (whiskers) of dorsal (c) and ventral (d) PCs grouped by locality and mating condition. *p*‐values of pairwise Tukey tests are indicated next to horizontal lines (locality comparisons) or on top of locality symbols (mating condition comparisons). Only significant effects in univariate analyses were tested with post hoc comparisons, for graphic simplicity only significant results are shown (see Tables S1, S2, S3 for detailed results)

Individual scores on the first two principal components derived from the dorsal spectral measurements (representing 84.4% of the variance in the data) indicated an overall trend toward higher values in Sarapiquí (Table S1, Figure 2a). MANOVA results indicated a significant effect of locality (Wilkinson's λ* *= 0.39, *F*_(4, 230)_ **= 34.88, *p *<* *.001), mating condition (Wilkinson's λ = 0.91, *F*_(2, 115)_ **= 5.63, *p *=* *.005) and the interaction between these two predictors (Wilkinson's λ = 0.85, *F*_(4, 230)_ **= 4.88, *p *<* *.001). No statistically significant effect of sex or any other interactions among predictors were observed. Univariate analysis of dorsal PC1 values (most correlated with brightness variables, see Table S1) indicated statistically significant differences among localities (*F*_(2, 116)_ **= 39.00, *p *<* *.001) and the interaction between localities and mating condition (*F*_(2, 116)_ **= 4.56, *p *=* *.012). Statistically significant differences in PC1 values were observed between Sarapiquí and the other two localities and within‐locality differences in mating condition were only significant in Sarapiquí where courting pairs exhibited higher values of PC1 (Figure 2c, Tables S2,S3) than single individuals. Analysis of dorsal PC2 values (most correlated with color variables, see SM Table 1) indicated a statistically significant effect of locality (*F*_(2, 116)_ **= 26.95, *p *<* *.001), mating condition (*F*_(2, 116)_ **= 11.28, *p *=* *.001), and the interaction between these two factors (*F*_(2, 116)_ **= 7.62, *p *<* *.001). Significant differences in dorsal PC2 values were observed between Isla Colón and the other two localities studied and differences in mating condition were only significant among individuals of Sarapiquí where courted individuals exhibit lower values of PC2 (Figure 2c, Tables S2, S3).

Analysis of the ventral measurements indicated a differentiation among localities with individuals from Sarapiquí attaining slightly higher values of the first two PC‐derived spectral variables (accounting for 85.9% of the total variance; Table S1, Figure 2b). MANOVA results indicated a statistical significant effect of locality (Wilkinson's λ = 0.43, *F*_(4, 230)_ **= 30.33, *p *<* *.001) but no effect of mating condition, sex, or any other interactions among predictors. Univariate analysis of ventral PC1 values (most correlated with brightness variables, see Table S1) showed statistically significant differences among the means of all three localities (*F*_(2, 116)_ **= 39.00, *p *<* *.001; Figure 2d, Tables S2, S3). Mean ventral PC2 values (most correlated with color contrast, see Table S1) differed significantly among localities (*F*_(2, 116)_ **= 8.92, *p *<* *.001) with individuals from Hitoy showing lower values than those from Sarapiquí and Colón (Figure 2d, Tables S2, S3).

## 4 DISCUSSION

The mechanisms that facilitate and maintain intraspecific color divergence among aposematic organisms remain an unresolved issue (Hegna, Galarza, & Mappes, 2015; Schaefer, Vences, & Veith, 2002) despite several in‐depth studies (Kapan, 2001). Some studies examining phenotypic variation in the strawberry poison frog have suggested that sexual selection promotes color divergence (Maan & Cummings, 2009; Richards‐Zawacki et al., 2012; Summers et al., 1999; Tazzyman & Iwasa, 2010), but the role of natural selection has also been discussed to drive divergence in this trait (Dreher, Cummings, & Pröhl, 2015; Maan & Cummings, 2012; Pröhl & Ostrowski, 2011). The most plausible scenario involves both natural selection (e.g., for initiation of color divergence), and sexual selection acting at different stages of divergence, and affecting different populations to a different degree (Cummings & Crothers, 2013; Dreher & Pröhl, 2014). Although studies investigating the importance of selection for phenotypic divergence in our study species are numerous, the importance of within‐population variation has received far less attention from the scientific community (Richards‐Zawacki et al., 2012) than among‐population divergence (Maan & Cummings, 2008; Pröhl & Ostrowski, 2011; Rudh, Rogell, Hastad, & Qvarnstrom, 2011).

Our results demonstrate that in a Costa Rican population (Sarapiquí), male and female frogs actively engaged in courtship have brighter dorsa, but lower values for coloration measurements, than individuals not engaged in these activities. Whether or not these differences between the groups were present before courtship or are a consequence of courtship itself is yet unknown. It is possible that courtship activities activate hormonal cascades in both sexes that influence coloration patterns. If these differences in brightness and coloration were present before courtship, then the observed nonrandom pairing may have some interesting implications for evolution of coloration patterns in this population.

These results confirm laboratory‐based estimates of female preferences for brighter males across several Panamanian populations of *O. pumilio* (Maan & Cummings, 2009). With their higher parental investment during reproduction, females are suggested to be the choosing sex in strawberry poison frogs (Pröhl & Hödl, 1999). Dorsal coloration is discussed to be the best visual indicator for mate selection (Maan & Cummings, 2008; Tazzyman & Iwasa, 2010), and within some populations, for example, on Isla Solarte, Panama, females exhibit a preference for brighter males (Dreher & Pröhl, 2014; Maan & Cummings, 2009). For the same population, sexual dimorphism with males being brighter than females has been reported (Maan & Cummings, 2009). Males also pay attention to rival brightness in this population and aggressive behavior of males is predicted by their own brightness (Crothers & Cummings, 2015; Crothers et al., 2011). Females might prefer brighter males because of their superior condition, competitive ability, or higher breeding experience (Bitton, Dawson, & Ochs, 2008; Murphy & Pham, 2012; Summers, Bermingham, Weigt, & McCafferty, 1997).

Female preferences for brighter colored males has been found in a diversity of other taxa including invertebrates (butterflies: Kemp, Jones, Macedonia, & Krockenberger, 2014; Kemp, Macedonia, Ball, & Rutowski, 2008; mantis: Barry, White, Rathnayake, Fabricant, & Herberstein, 2015) as well as vertebrates (toads: Vasquez & Pfennig, 2007; birds: Loyau et al., 2007; but see Beausoleil, Doucet, Heath, & Pitcher, 2012 for no such preferences in red‐side dace). Only few studies report on the benefits that females gain by mating with brighter colored males. For the spadefoot toad (*Scaphiopus couchii*), females seem to use coloration brightness to identify larger males in better condition (Vasquez & Pfennig, 2007). In a beetle species, higher brightness is correlated with enhanced predator avoidance because brightness indicates better deterrence against predators (Bezzerides, McGraw, Parker, & Husseini, 2007). Furthermore, some evidence suggests that more colorful males, and sometimes females, are more likely to win fights and obtain and defend high‐quality territories (review in Rojas, 2017).

Our results suggest that in one population of *O. pumilio* male phenotype affects mating success and very likely reproductive success, with sexual selection as a possible driver for divergence in this trait. However, the fact that brightness measurements were significantly higher while color indices were significantly smaller for courting males needs further attention. Mechanisms that promote elevated brightness (within populations) are discussed to have the potential to cause shifts in hue as well and might have influenced divergence in coloration among different populations of this species (Maan & Cummings, 2009). Additional studies will help to clarify whether there might be a trade‐off between color and brightness contrasts in poison‐dart frog coloration. Interestingly, the absence of an effect of sex on any of these comparisons indicates that differences in color and brightness also exist between females from Sarapiquí engaged in courtship and those not engaged in courtship.

In *O. pumilio,* females also defend small territories most likely associated with food resources (Meuche, Linsenmair, & Pröhl, 2011; H. Pröhl et al. unpublished data) and brighter females also might be more competitive and in better condition. In case several females arrive at the territories of the males these might court the brightest females most intensely, while the other females leave the territory without entering courtship. On the other hand, it might be possible that less bright females avoid brighter males because of the enhanced probability to be detected by a predator (Marzal et al., 2016). Whether an extremely bright and conspicuous coloration as observed in Sarapiquí and Solarte can evolve might depend on resource availability and the heritability of coloration traits. There is evidence that the color of the skin is heritable in *O. pumilio* (Summers et al., 2004), but the heritability of brightness is unknown. Based on some theoretical models, Lee, Speed, and Stephens (2011) discuss the possibility of bright conspicuous coloration in combination with high toxicity to emerge in aposematic species when resources (toxins and pigments) are abundant in the habitat and when additional benefits for bright coloration such as mate choice are present. Future studies testing the relationship between toxicity, coloration brightness, and mating success across a broader range of strawberry poison frog populations would be an excellent test of this prediction. For *O. pumilio,* a recent study further demonstrated that more toxic and conspicuous frogs produced fewer tadpoles because of poorer physiological or behavioral tadpole performance (Dugas & Richards‐Zawacki, 2015). In such cases, adults might compensate tadpole loss by higher mating activities (Pröhl, 2005).

Many dendrobatid species use aposematic coloration to display their toxicity to potential predators. Across Panamanian populations of strawberry poison frogs in the Bocas del Toro archipelago, including various distinct color morphs, brightness has been found to be an honest indicator of toxicity (Maan & Cummings, 2012). A recent study (Cummings & Crothers, 2013) suggests that low toxicity levels constrain populations to a cryptic coloration, while high toxicity levels provide protection against predators and therefore allows for coloration to be shaped by sexual selection. Here, we show that strawberry poison frogs from the Costa Rican Sarapiquí population have higher dorsal reflectance than those from Hitoy Cerere and Isla Colón (Figure 1) and that couples in the Sarapiquí population show higher color brightness than single individuals. In the aposematic species *Dendrobates tinctorius,* movement behavior is related to the color pattern of individuals (Rojas, Devillechabrolle, & Endler, 2014). It is therefore possible that in *O. pumilio* brighter males and females, which could be more toxic, exhibit a different behavior conferring higher mating success than that of duller individuals. Under this rationale, brighter individuals are more toxic and better protected which allows for being more active, faster, calling more, defending better territories, visiting more exposed sites and therefore be able to attract mates of similar phenotype.

Some animal species are able to undertake rapid color changes, which may be employed in intra‐ and interspecific communication, for example, for predator deterrence, territorial fights, and courtship behaviors (Hanlon, 2007; Stuart‐Fox & Moussalli, 2008). Beside rapidly changing color patterns, well known for cephalopods and chameleons for example, some species temporarily exhibit certain color patterns during courtship or breeding seasons. In zebrafish (*Danio rerio*), both sexes change their striping pattern during spawning (Hutter, Hettyey, Penn, & Zala, 2012). Body coloration of the male moor frogs (*Rana arvalis*) changes from dull brown to bright blue during the short breeding season, resulting in a temporal sexual dichromatism, which probably facilitates sex discrimination in this explosive breeder (Sztatecsny et al., 2012). Dynamic and drastic changes of coloration in males are also known in some other explosive breeders during the reproductive period (Aspengren, Sköld, & Wallin, 2009; Bell & Zamudio, 2012; Rudh et al., 2013; Toledo & Haddad, 2009). Changes in body coloration in amphibians are well documented and controlled by a diverse set of hormones (Sköld, Aspengren, & Wallin, 2013). Hormone‐controlled aggregation or dispersion of pigment containing organelles in chromatophores in the frog's skin cause lightening or darkening of the skin, respectively. These processes are best understood in cryptic species which can rapidly change their coloration to improve background matching. However, no significant short‐term color changes have been reported in aposematic prolonged breeders like *O. pumilio* neither in the context of reproduction, nor predator avoidance. Whether strawberry poison frogs alter their brightness or coloration during courtship, which could be an alternative explanation for the observed differences between courting and single animals in Sarapiquí, cannot be detected with the method applied in this study and thus warrants further attention. Skin brightness could be correlated with reproductive hormone levels; but a recent study with male *O. pumilio* on Solarte (Panama) found no correlation between male dorsal brightness and circulating testosterone levels (Crothers et al., 2016).

In this study, we measured the reflectance of courting couples, which were found in a late stage of courtship. Interruption of courtship during this stage is very rare, and due to external reasons (e.g., disturbance by other animals, pers. obs.). Once a female follows a calling male to the oviposition site, egg deposition is very likely, and reproductive success seems to be positively correlated with mating frequency under most circumstances (Pröhl, 2005). Our study is the first to provide field evidence that brighter individuals might have greater reproductive success than duller ones in some populations of *O. pumilio*. Our findings from the population in Sarapiquí, together with the sexual dimorphism and preference for brighter males in the population of Solarte (Maan & Cummings, 2009), suggest that sexual selection affects brightness only in some, but not all populations of strawberry poison frogs. Furthermore, our results suggests that sexual selection on coloration is not restricted to Bocas del Toro populations (Southern genetic group), where color divergence among populations is remarkable, but also operates in Costa Rica (Northern genetic group), where all populations are red on the dorsum (Hagemann & Pröhl, 2007). With color being affected by natural selection (predator avoidance) and sexual selection (intra‐ and intersexual selection), and females having differential preferences for local coloration and brighter males (Maan & Cummings, 2008, 2009; Reynolds & Fitzpatrick, 2007; Summers et al., 1999), coloration of strawberry poison frogs might thus be a classic magic trait. A trait considered magic is subject to both divergent ecological selection and nonrandom mating (Servedio, van Doorn, Kopp, Frame, & Nosil, 2011). In *O. pumilio,* increased local availability of natural toxins might allow for selection toward higher toxicity and higher dorsal brightness and females might prefer better adapted, that is, brighter and more toxic males at these places. Interactions between sexual and predator selection for shaping the local phenotype has been proposed for several aposematic Neotropical poison frogs and butterflies (Merrill et al., 2012; Noonan & Comeault, 2009; Rudh et al., 2011). This interplay of selective forces possibly contributes to the emergence of phenotypic variation within and among populations of brightly colored aposematic animals, including strawberry poison frogs.

## CONFLICT OF INTEREST

None declared.

## AUTHOR CONTRIBUTIONS

C.E.D. and H.P. designed the research. C.E.D. performed field work, C.E.D., A.R. and M.E.C. analysed the data. C.E.D., H.P and A.R. wrote the paper. All authors revised the manuscript and approved the final version.
