# Supplementary material for: Mating status correlates with dorsal brightness in some but not all poison frog populations
Source: Ecol Evol. 2017 Nov 1;7(24):10503–12. doi: 10.1002/ece3.3531 (PMC5743646; doi:10.1002/ece3.3531)
Supplement: Supplementary file 1 [file ECE3-7-10503-s001.docx]

Supplementary Material Table 1. Variable correlations and variance explained by the principal components extracted from the dorsal and ventral spectral reflectance measurements of 131 individuals of *Oophaga pumilio*. See main text for a description of the variables and their abbreviations.

|  | PC1 | PC2 | PC3 | PC4 | PC5 |
| --- | --- | --- | --- | --- | --- |
| Dorsal measurements |  |  |  |  |  |
| ΔS | -0.14 | **0.91** | -0.10 | 0.38 | 0.00 |
| ΔL | **0.84** | 0.08 | 0.53 | 0.10 | 0.02 |
| S | -0.07 | **0.92** | 0.09 | -0.38 | 0.01 |
| L | **0.96** | 0.10 | -0.20 | -0.03 | -0.18 |
| T | **0.95** | 0.04 | -0.28 | -0.03 | 0.17 |
| *eigenvalue* | *2.5* | *1.7* | *0.4* | *0.3* | *0.1* |
| *variance (%)* | *50.6* | *33.8* | *8.4* | *6* | *1.2* |
| *cumulative variance (%)* | *50.6* | *84.4* | *92.8* | *98.8* | *100* |
| Ventral measurements |  |  |  |  |  |
| ΔS | -0.55 | **0.77** | 0.02 | -0.33 | 0.05 |
| ΔL | **0.76** | 0.42 | -0.49 | 0.06 | 0.01 |
| S | -0.68 | 0.64 | 0.07 | 0.36 | -0.02 |
| L | **0.87** | 0.36 | 0.22 | -0.04 | -0.23 |
| T | **0.91** | 0.23 | 0.26 | 0.06 | 0.23 |
| *eigenvalue* | *2.93* | *1.37* | *0.36* | *0.24* | *0.11* |
| *variance (%)* | *58.6* | *27.3* | *7.2* | *4.8* | *2.1* |
| *cumulative variance (%)* | *58.6* | *85.9* | *93.1* | *97.9* | *100.0* |

**Supplementary Material Table 2.** Results of the Tukey HSD post-hoc test of univariate ANOVA analyses of PC1 (upper-diagonal) and PC2 (lower diagonal) scores derived from spectral measurements in *Oophaga pumilio*. In each cell, the P-values of dorsal (top) and ventral (bottom) comparisons of locality means are provided, significant results are bolded.

|  | Sarapiquí | Hitoy | Colón |
| --- | --- | --- | --- |
| Sarapiquí |  | **< 0.001**  **< 0.001** | **< 0.001**  **< 0.001** |
| Hitoy | 0.057  **< 0.001** |  | 0.998  **0.003** |
| Colón | **< 0.001**  0.285 | **< 0.001**  **0.028** |  |

**Supplementary Material Table 3**. Results of the Tukey HSD post-hoc test of univariate ANOVA analyses of PC scores derived from spectral measurements in *Oophaga pumilio*. In each cell, the P-values of comparisons of the PC scores means for single vs courtship individuals per locality are provided, significant results are bolded.

| Surface | PCs | Sarapiquí | Hitoy | Colón |
| --- | --- | --- | --- | --- |
| Dorsal | PC1 | **0.025** | 0.986 | 0.999 |
|  | PC2 | **< 0.001** | 0.618 | 0.997 |
| Ventral | PC1 | 0.467 | 0.932 | 1.000 |
|  | PC2 | 1.000 | 0.996 | 0.673 |
